# The Natural Compound Oblongifolin C Exhibits Anticancer Activity by Inhibiting HSPA8 and Cathepsin B *In Vitro*

**DOI:** 10.3389/fphar.2020.564833

**Published:** 2020-12-17

**Authors:** Li Han, Danqing Xu, Zhichao Xi, Man Wu, Wan Najbah Nik Nabil, Juan Zhang, Hua Sui, Wenwei Fu, Hua Zhou, Yuanzhi Lao, Gang Xu, Cheng Guo, Hongxi Xu

**Affiliations:** ^1^ School of Pharmacy, Shanghai University of Traditional Chinese Medicine, Shanghai, China; ^2^ Department of Pharmacy, Shanghai Jiao Tong University Affiliated Sixth People's Hospital, Shanghai, China; ^3^ School of Chinese Medicine, Faculty of Medicine, The Chinese University of Hong Kong, Hong Kong, China; ^4^ Institute of Cardiovascular Disease of Integrated Traditional Chinese and Western Medicine, Shuguang Hospital, Shanghai University of Traditional Chinese Medicine, Shanghai, China; ^5^ State Key Laboratory of Phytochemistry and Plant Resources in West China and Yunnan Key Laboratory of National Medicinal Chemistry, Kunming Institute of Botany, Chinese Academy of Sciences, Kunming, China

**Keywords:** natural compounds, oblongifolin C, HSPA8, cathepsin B, protein fishing

## Abstract

PPAPs (Polycyclic polyprenylated acylphloroglucinols) are a class of compounds with diverse bioactivities, including anticancer effects. Oblongifolin C (OC) is a PPAP isolated from the plant of *Garcinia yunnanensis* Hu. We previously discovered that OC induces apoptosis, inhibits autophagic flux, and attenuates metastasis in cancer cells. However, the protein targets and the detailed mechanism of action of OC remain unclear. To identify protein targets of OC, a non-labeled protein fishing assay was performed, and it was found that OC may interact with several proteins, including the heat shock 70 kDa protein 8 (HSPA8). Expanding on our previous studies on protein cathepsin B, this current study applied Surface Plasmon Resonance (SPR) and Isothermal Titration Calorimetry (ITC) to confirm the potential binding affinity between OC and two protein targets. This study highlights the inhibitory effect of OC on HSPA8 in cancer cells under heat shock stress, by specifically inhibiting the translocation of HSPA8. OC also enhanced the interaction between HSPA8, HSP90, and p53, upregulated the expression of p53 and significantly promoted apoptosis in cisplatin-treated cells. Additionally, a flow cytometry assay detected that OC sped up the apoptosis rate in HSPA8 knockdown A549 cells, while overexpression of HSPA8 delayed the OC-induced apoptosis rate. In summary, our results reveal that OC potentially interacts with HSPA8 and cathepsin B and inhibits HSPA8 nuclear translocation and cathepsin B activities, altogether suggesting the potential of OC to be developed as an anticancer drug.

## Introduction

Natural compounds from medicinal plants are important resources in developing drugs for a wide variety of diseases, including cancer, neurodegenerative diseases, depression, and infections (Newman and Cragg, 2016; Salehi et al., 2019). Polycyclic polyprenylated acylphloroglucinols (PPAPs), are a class of compounds that are mainly extracted from *Guttiferae* and related plant families. PPAPs are composed of a highly oxygenated and densely substituted bicyclo[3.3.1]nonane-2,4,9-trione or bicyclo[3.2.1]octane- 2,4,8-trione core connected to C5H9 or C10H17 (prenyl, geranyl, etc.) side chains (the latter exists in several isomeric forms). Interestingly, the complex structure and bioactivities of PPAPs have attracted substantial attention from both biologists and chemists in recent years (Ciochina and Grossman, 2006) and the mechanism of anticancer action of PPAPs have recently been extensively investigated (Yang et al., 2018). Oblongifolin C (OC) is a PPAP isolated from the plant of *Garcinia yunnanensis* Hu. Our earlier studies elucidated the mechanism of action of OC on tumor growth by using various cell based screening systems and revealing that OC has profound anticancer activities, such as inducing apoptosis and inhibiting autophagic flux and metastasis in different types of cancer cells (Feng et al., 2012; Lao et al., 2014; Wang et al., 2015). However, the mechanism of action of OC is not fully delineated and remains unclear; in particular, the protein targets of OC in cancer cells have yet to be identified to support its further development as a potent anticancer drug.

In fact, elucidating the protein targets of active compounds represents a key obstacle in developing drugs from natural compounds (Moumbock et al., 2019), with only a few successful natural compounds having identified direct targets. For instance, paclitaxel (Taxol) is a complex taxane diterpene isolated from the Pacific yew tree four decades ago and is currently used as a chemotherapy for various tumors, such as lung, breast, and ovary tumors (Bakrania et al., 2016). Paclitaxel binds to the *β*-subunit of tubulin, resulting in cytoskeleton framework distortions, G2/M cell cycle arrest, and subsequent cell death in cancer cells. However, the protein targets of other effective natural compounds, such as artemisinin, triptolide, celastrol, and curcumin are still under investigation (Corson and Crews, 2007). Triptolide, a diterpenoid epoxide, and celastrol, a pentacyclic triterpene, are isolated from the traditional Chinese medicine *Trypterygium wilfordii Hook* F. These two compounds exhibit a staggering variety of cellular effects, including anticancer, obesity, and inflammatory effects. Triptolide strongly inhibits transcriptional activity, possibly by targeting NF-κB and RNA polymerase II (Wang et al., 2011; Zong et al., 2019). Celastrol increases leptin sensitivity and suppresses food intake in obese mice through an unknown molecular mechanism (Liu et al., 2015). Artemisinin, an antimalarial drug isolated from *Artemisia annua* L, interacts with several proteins, such as TCTP, SERCA orthologue PfATP6, and *P. falciparum* cysteine proteases (Diedrich et al., 2018). However, the described interactions between artemisinin and other proteins may not accurately explain its anticancer effects. Thus, scientists are still working to examine compound-target interactions using multiple techniques. With the rapid development of genomic and proteomic techniques, computational modeling has become a popular method for predicting compound-protein interactions (Cichonska et al., 2015; Keum et al., 2016; Tsubaki et al., 2019). Although there are several database services, predicting interactions among proteins still requires laborious wet lab experiments to narrow down the possible interactions. The drug target fishing technique is another option for identifying drug targets (Eichhorn et al., 2012; Saxena, 2016). However, this method utilizes linker-modified compounds to perform protein pulldowns and tagged molecules may lose their binding activities as well as other chemical characteristics, leading to many false positive results. Using unlabeled molecules to pull down binding proteins address these concerns. Efferth et al. managed to identify novel binding proteins of artemisinin from nasopharyngeal cancer cells using PolySorp immunotubes (Eichhorn et al., 2012). In the present study, we focused on novel binding proteins of OC. The strong hydrophilic properties of OC permitted us to coat PolySorp immunotubes and to perform pulldowns without protein labeling. Our results indicated that OC potentially interacts with several proteins, including heat shock 70 kDa protein 8 (HSPA8). Using multiple chemical biology techniques, we confirmed the potential interaction between OC and HSPA8 and further extended our previous findings of OC on protein cathepsin B (Cath B) (Lao et al., 2014). Furthermore, we showed that OC regulates the function of HSPA8 in several cancer cell lines. In summary, our study explores the underlying anticancer mechanisms of the natural compound OC.

## Materials and Methods

### Materials

Oblongifolin C (>95% purity) was isolated from the fruits of *Garcinia yunnanensis* Hu using the same preparation method as previously described (Wang et al., 2016; **Supplementary Figures S1–S4; Table S1**). The plants were collected at Dehong, Yunnan, People’s Republic of China in August 2015 and identified by Dr Guo-Dong Li (Yunnan University of Traditional Medicine) by referring to the voucher specimen (KUN 0941149) that was deposited at the Kunming Institute of Botany, Chinese Academy of Science. We procured 2-Phenylethynesulfonamide (PES, >95% purity) from Sigma-Aldrich (Cat # P0122-5MG) and Cisplatin from Selleck (Cat # S1166, >99% purity). They were, respectively, dissolved in dimethyl sulfoxide (DMSO) and dimethylformamide (DMF) to produce a stock concentration of 10 mM and were stored at −20°C. We purchased a FITC Annexin V PE Apoptosis kit from BD Biosciences (Cat # 556,547), Cell Counting Kit-8 (CCK-8) from Dojindo (Cat # CK04) and 4′,6-diamidino-2-phenylindole hydrochloride (DAPI) from Sigma-Aldrich (Cat #D9542). We purchased recombinant protein Lysozyme C (Cat #: CI91), Alpha-enolase (Cat #: CR49), Caspase-14 (Cat #: CG19) from Novoprotein company (Shanghai, China). HSPA2 (Cat #: H00003306-P01) from Amyjet Scientific company (Shanghai, China), Histone deacetylase 4 (HDAC4, Cat #: 11807-HNCB) from Sino Biological (Beijing, China).

### Fishing of Potential Oblongifolin C Binding Proteins

**FIGURE 1:**
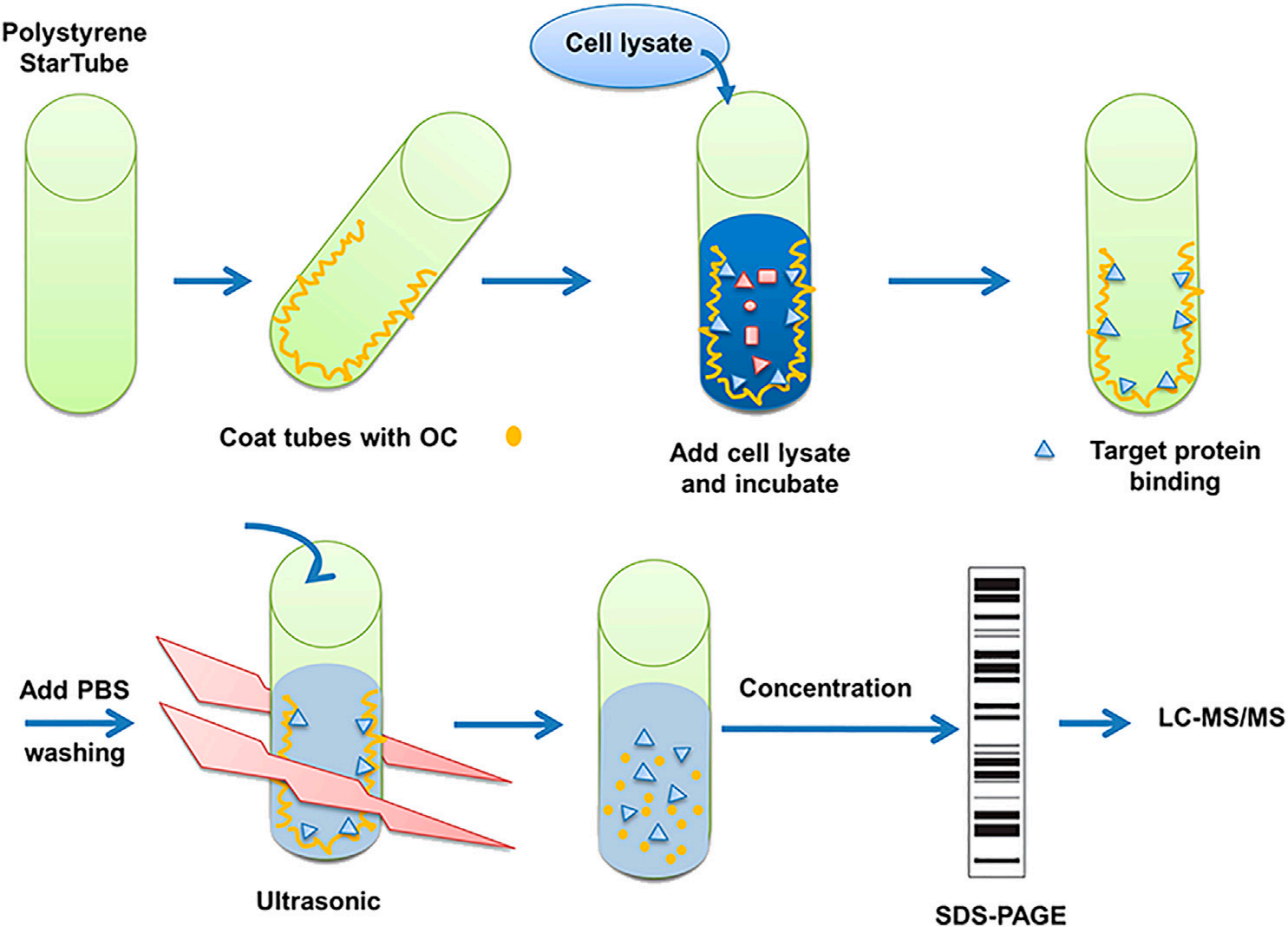
Description of method to search for potential interaction partners of OC. Fishing assay was first employed using OC-coated tubes, with vehicle (99.9% ethanol) incubated tubes serving as the control. HeLa cell lysates were then added to identify proteins that potentially bind to OC. Bound proteins were isolated, digested with trypsin, and identified by mass spectrometry.

The detailed OC binding and protein fishing flowchart is shown in Figure 1. Briefly, OC was attached to the surface of the PolySorp immunotube (Sigma-Aldrich, United States), with vehicle (99.9% ethanol) incubated tubes serving as a negative control. 2 mg OC was first dissolved in 5 ml of ethanol (99.9%), then the compound solution was transferred to an immunotube, and ethanol was removed using a vacuum pump at room temperature. The protein was sourced from 2 × 10^6^ HeLa cells. The cells were lyzed with 2 ml of RIPA buffer (Beyotime, China) supplemented with a protease inhibitor mix (Roche, Mannheim, Germany) for 30 **min at** 4°C, followed by a 30 min centrifugation at 14,000 rpm and 4°C to remove the undissolved proteins and cell debris. The soluble proteins were transferred to OC-coated tubes, which were shaken at 60 rpm at 4°C overnight (RIPA buffer was exposed to OC-coated immunotube to serve as another negative control). The tubes were washed thrice with phosphate-buffered saline (PBS). Bound proteins were eluted in 5 ml of PBS by sonication for 30 min at 4 °C. The whole solution was transferred to an Eppendorf reaction tube and concentrated to a final volume of 100 µl. The proteins were subjected to SDS-PAGE gel electrophoresis. The confirmed protein bands of three independent experiments were retrieved, digested with trypsin and sent to the APPLIED PROTEIN TECHNOLOGY (Shanghai, China) for proteomics analysis using LC-MS/MS. LC-MS/MS analysis was performed on a Q Exactive mass spectrometer (Thermo Scientific) and UPLC-ESI-MS/MS was conducted as per an earlier protocol (Eichhorn et al., 2012). MS/MS spectra were searched using the MASCOT engine (Matrix Science, London, United Kingdom) against the Uniprot database. For protein identification, the following settings were applied; peptide mass search tolerance at 20 ppm, MS/MS tolerance at 0.1 Da, and Minimal score at 20.

### Protein Expression and Purification

Full-length HSPA8 (pcDNA5/FRT/TO HIS HSPA8, Addgene, Cat # 19,541) or cathepsin B (hCathepsin B, Addgene, Cat # 11,249) was subcloned into the pET-15b vector *via Nco*I-*BamH*I sites. his_HSPA8 and his_hCathepsin B were expressed in *Escherichia coli* BL21 (DE3) cells, respectively. After cell lysis, the protein was purified by affinity chromatography using a His Trap_HP column (GE Healthcare Biosciences). Further purification was performed using a Q_HP column (GE Healthcare Biosciences), followed by Superdex 75 columns (GE Healthcare Biosciences). Moreover, the protein was concentrated to 2 mg/ml in 20 mM HEPES, 150 mM NaCl, and 0.5 mM tris (2-carboxyethyl) phosphine (TCEP) (pH 7.4).

### Surface Plasmon Resonance Assay

Analysis of potential binding between his_HSPA8 or his-cathepsin B and OC was performed with a Surface Plasmon Resonance (SPR) biosensor (Biacore T200, GE Healthcare). The surface of the CM5 sensor chip was activated by injecting a solution containing 200 mM EDC and 50 mM NHS at 10 μl/min for 420 s his_HSPA8 (0.2 mg/ml, 200 µl) or his_cathepsin B (0.2 mg/ml, 200 µl) in 10 mM sodium acetate buffer (pH 4.5) was immobilized to the CM5 chip *via* an amine coupling reaction. The surface was then blocked by injecting 1 M ethanolamine-HCL for 420 s his_HSPA8 or his_cathepsin B was immobilized at approximately 10,000 Response Unit (RU) to channel 2 of CM5 chip. Channel 1 was used as a blank control. Binding analysis was performed in an HBS-EP buffer with 1% DMSO using a flow rate of 30 μl/min at 25°C. Various concentrations of OC were successively injected upon immobilization of his_HSPA8 protein to detect the SPR response-generated binding using a flow rate of 30 μl/min for 90 s.

### Isothermal Titration Calorimetry Assay

An isothermal titration calorimetry (ITC) assay was performed in a Microcal iTC200 instrument (Malvern, United Kingdom). All protein samples were dialyzed in a 20 mM HEPES buffer with 200 mM NacL and 5% DMSO, pH 7.5. The experiments were carried out in 25°C. 25 µM his_HSPA8 or his_cathepsin B were placed in sample cells. 500 µM small molecular OC was placed in the syringe. Small molecular OC titrated protein samples using a syringe with 19 successive additions of 2 µl for 4 s with 750 rpm stirring. Titration curves were fitted using one set of sites model with origin software supplied with the Malvern Microcal iTC200 instrument.

### Thermal-Shift Assay (ThermoFluor)

The thermal shift assay was performed using the ViiA™ seven Real-Time PCR System (Thermo Fisher Scientific, Inc.), which was originally designed for PCR. The thermal stability of HSPA8 was detected using Protein Thermal Shift™ Dye Kit (Cat# 4,461,146, Life Technologies). To identify compounds that bind to HSPA8, a dye that binds to the exposed hydrophobic residues was employed. A temperature increment of 1°C/min was applied. Samples contained 1 µg of HSPA8, OC, or PES (12.5 µM) and 0.5% DMSO with total volume of 20 µl in 1X Protein Thermal Shift™ Buffer.

### Cell Lines and Cell Culture

A549 cells were purchased from the Shanghai Institute of Cell Biology, Chinese Academy of Sciences (Shanghai, China). HeLa cells with stable expression of GFP-LC3 was a gift from Professor Naihan Xu in Tsinghua University. Other cell lines were purchased from American Type Culture Collection (ATCC). Cells were maintained at 37°C with a 5% CO_2_ humidified atmosphere in growth medium as recommended by the vendors; cells lines that were subjected to assays were between passages 8–15.

### Cell Proliferation Assay

Each cell line was seeded in a 96-well tissue culture plate (Corning, NY, United States) at a predetermined density and cultured overnight in 180 µl of complete medium. On the next day, the cells were treated with natural products or compounds for another 72 h. Then, the medium was discarded and replaced with 10% CCK-8 (Dojindo, Kumamoto, Japan) in complete medium, and the plates were incubated for another 2 h. OD450 was measured with a SpectraMAX 190 spectrophotometer (MDS, Sunnyvale, CA). The background absorbance of the OD blank was subtracted from all of the wells. The inhibition rate (IR) was determined with the following formula: IR (%) = (OD_DMSO_−ODcompound)/OD_DMSO_×100%. The CCK-8 assay as above was used to determine cell viability.

### Propidium Iodide and Annexin V Double Staining for Flow Cytometry

A549 cells were collected and washed with cold PBS buffer twice after digestion with trypsin. 3 × 10^5^ cells were resuspended with 100 µl binding buffer (BD Biosciences Cat # 556,547), and then stained with 5 µl PI/Annexin V (Sigma) for 15 min in the dark. Then, the apoptotic cells were detected using a FACSCalibur (BD, United States).

### Western Blotting

The cell lysate was prepared in RIPA buffer and quantified by the bicinchoninic acid (BCA) method (Pierce, Rockford, IL, United States). Ten to 30 µg of total protein per sample was loaded onto a 4–12% NuPAGE^®^ Novex SDS gel (Invitrogen). The protein was transferred by an iBlot^®^ dry blotting device (Invitrogen) onto nitrocellulose membranes. After blocking the nonspecific binding with TBS/Tween 20 (0.1%) (TBS/T) containing 5% nonfat milk for 1 h at room temperature (RT), the membrane was incubated with primary antibodies of LC3B (Sigma, L7543), SQSTM1/p62 (MBL, PM045), PARP (Cell Signaling Technology, CST # 9542P), or Caspase-3 (Cell Signaling Technology, CST # 9662P) (1:1,000 diluted in TBS/T containing 3% bovine serum albumin (BSA) and gently shaken at 4°C overnight. The membrane was washed with TBS/T thrice to remove the unbound antibody and then incubated with the secondary antibodies (HRP-conjugated goat anti-mouse IgG or goat anti-rabbit IgG, 1:5,000, KangChen Biotech, Shanghai, China) for 1 h at RT. Protein bands were visualized with an enhanced chemiluminescence (ECL) kit (Pierce, Rockford, IL, United States). The intensities of the selected bands were analyzed by ImageJ software.

### Pulldown Assay to Identify Potential Interaction Between Oblongifolin C and HSPA8

For OC and HSPA8 potential interaction detection, A549 cell lysates were used. The A549 cell lysate was prepared and quantified by the BCA method. Briefly, 100 µg of total protein was suspended in IP lysis buffer (Pierce™ IP Lysis Buffer, Cat # 87,788), which were respectively added to biotin, OC or OC-biotin (20 µM) for incubation overnight at 4°C. After incubation, 10 µl of Avidin Resin (Pierce, Cat # 53,146) was added and incubated for another 2 h, and then washed four times with an IP lysis buffer. Samples were added with a loading buffer and analyzed by SDS-PAGE. The HSPA8 protein was detected using the anti-HSPA8 antibody by western blotting.

### Immunofluorescence of HSPA8 Translocation and Quantitative Analysis

A549 cells were seeded into two 96-well plates (clear bottom, black; PerkinElmer) and cultured overnight. Cells were pretreated with different concentrations of OC or OC-biotin for 1 h. Afterward, one plate was moved to a 42°C incubator with a 5% CO_2_ humidified atmosphere, and the other plate was left to incubate at 37°C. After 4 h incubation, the cells were washed with PBS, fixed with 4% paraformaldehyde for 15 min and blocked with a blocking buffer (0.1% Triton X-100, 1% BSA and 10% FBS in PBS) for 1 h. Cells were then incubated with anti-HSPA8 antibody (Sigma, Cat # SAB2701964–100UL) 1:200 diluted in a blocking buffer at 4°C overnight. After washing twice with PBS, cells were incubated with Alexa Fluor 488 conjugated goat anti-rat IgG antibody (Invitrogen, Cat # A-11006) (1:500 dilution) for 1 h at room temperature. Cells were washed twice with PBS and incubated with DAPI for 5 min. Image acquisition was performed by an Opera High Content Screening System (Perkin Elmer). The data were analyzed by the software Columbus 2.3 (Perkin Elmer instrument). To quantify HSPA8 translocation from the cytoplasm to the nucleus, the following procedures were performed: 1) The Hoechst channel was used to define the nuclear region (method B; common threshold 0.2 with area>30 μm); 2) The Alexa 488 channel was used to define the cellular cytoplasm region (method B; individual threshold 0.4); and 3) The Alexa 488 channel was used to calculate the intensity properties of both the nuclei and cytoplasm.

### Transfection

A549 cells were seeded into a six-well plate with a density of 3 × 10^5^ cells/well. On the next day, medium was changed before transfecting cells with siRNA (Merck, Cat # EHU115141) using Lipofectamine RNAiMAX Reagents (Invitrogen, Cat # 13,778–075) according to the manufacturer's protocols. The HSPA8 gene was subcloned into pCDNA3.1 vector, the pCDNA_HSPA8 plasmid was transfected with Lipofectamine 3,000 (Invitrogen, Cat # L3000–015).

### Statistical Analysis

Data were analyzed with GraphPad Prism five software and expressed as mean ± standard deviation (SD). One-way analysis of variance (one-way ANOVA) was used to examine the variance among different groups. *p value <0.05 was considered a statistically significant difference.

## Results

### Fishing and Identifying of Oblongifolin C Potential Binding Proteins

**FIGURE 2:**
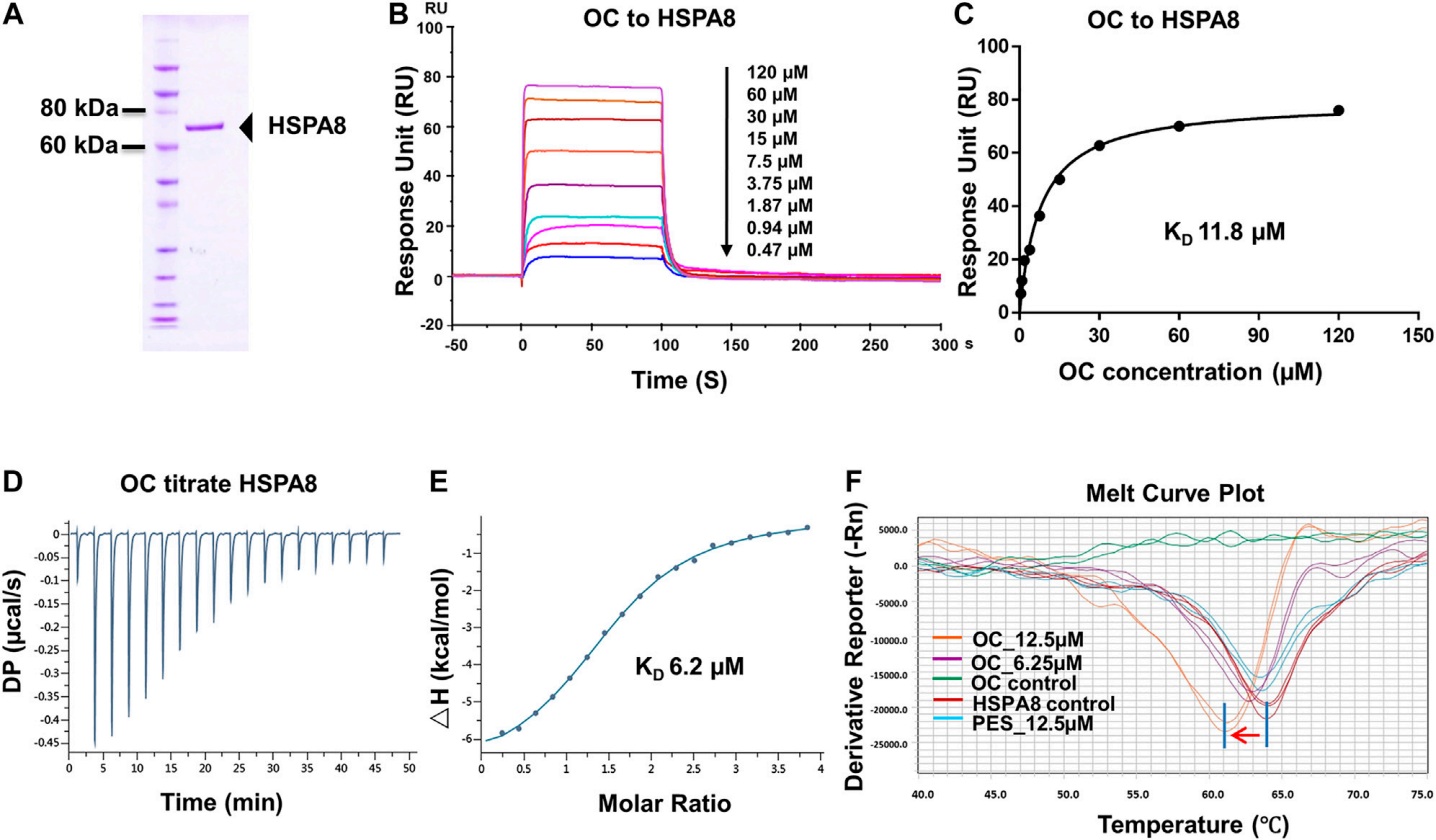
Verifying the potential binding of HSPA8 with OC. A Coomassie blue staining of HSPA8. **(A)** total of 3 µg of purified HSPA8 was loaded for SDS-PAGE followed by Coomassie blue staining. **(B)** Biacore SPR analysis of HSPA8 and OC binding. HSPA8 was immobilized on the CM5 chip and different concentrations of OC passed through the chip for 60 s. The curves indicate the potential binding between OC and HSPA8. **(C)** The KD value was calculated from Biacore SPR data. **(D)** ITC assay of HSPA8 and OC was performed at 25°C. The raw ITC data for injecting OC into the sample cell containing HSPA8 protein. The reaction heat of OC and protein binding was expressed as differential power (DP) between the reference and sample cells. **(E)** Experimental data are expressed with solid dots and fitted to a binding curve by a one-site binding model. **(F)** Thermal shift assay of HSPA8 and OC. HSPA8 was incubated with different concentrations of OC, and the melting curve was plotted and calculated using fluorescence intensity.

To fish for potential binding proteins using unlabeled OC, we similarly followed a protocol by Efferth's group, as described in the materials and methods section (Figure. 1; Eichhorn et al., 2012). Briefly, the cell lysates were incubated with OC-coated immunotubes. After sonication, concentration, gel electrophoresis and digestion, the bound proteins were analyzed by LC-MS/MS. Of 152 identified potential proteins bondable to OC (**Supplementary Table S2**), we performed SPR assay on seven apoptosis- and autophagy-related potential proteins to compare their binding affinity with OC. The examined potential proteins included Lysozyme C, Alpha-enolase, Caspase-14, Histone deacetylase 4 (HDAC4), Cathepsin B, HSPA8, and HSPA2. Both HSPA8 and HSPA2 belong to Heat Shock Protein 70 (HSP70) family, and they are motifs and structurally similar. SPR data showed that HSPA8 and Cathepsin B had a strong binding affinity with OC (Figures 2BC, 3BC; **Supplementary Figure S5**). We chose HSPA8 and Cath B as candidates for further studies.

**FIGURE 3:**
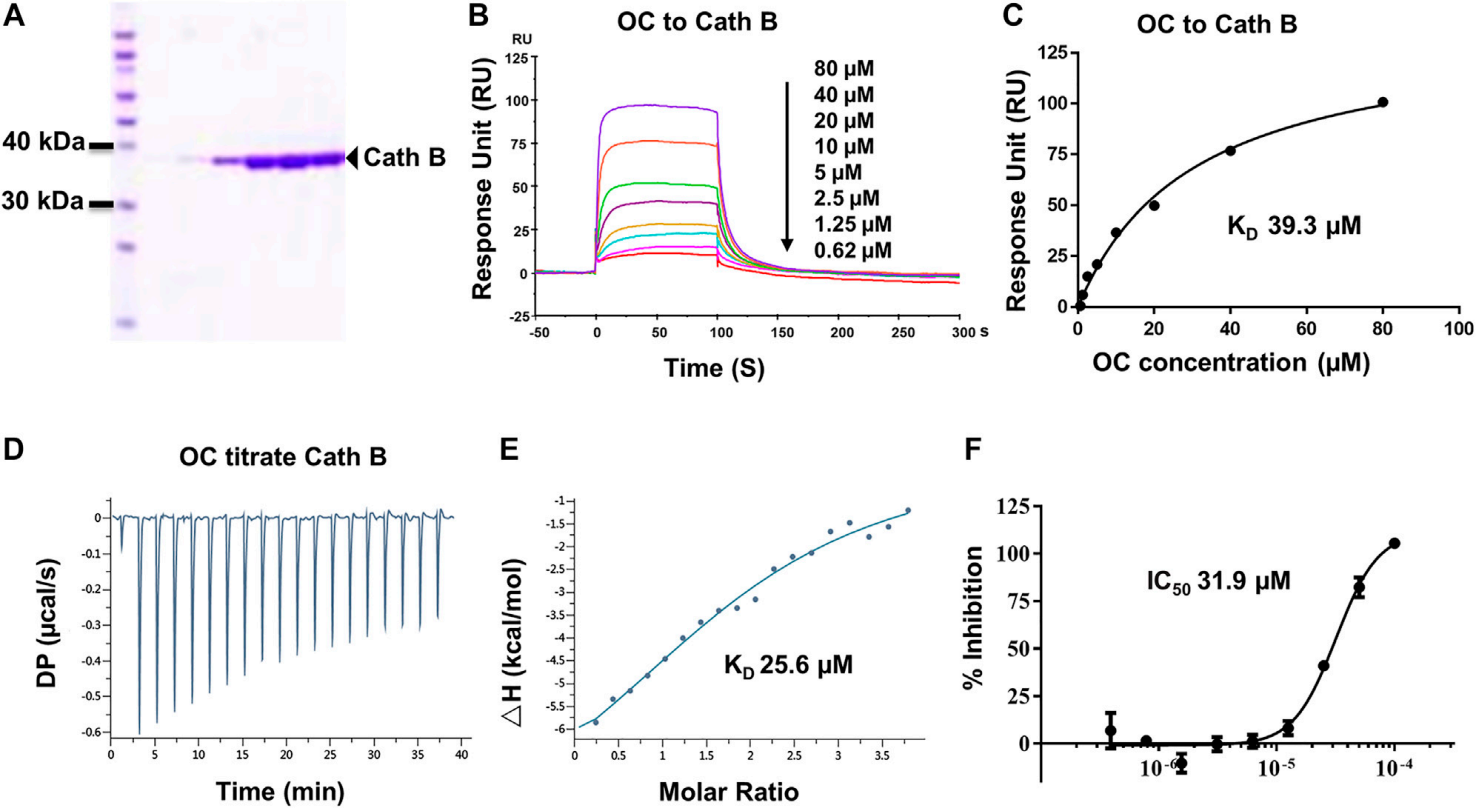
Verifying the potential binding of Cath B with OC. A Coomassie blue staining of Cath B. **(A)** total of 10 µg of Cath B purified from different conditions were loaded for SDS-PAGE followed by Coomassie blue staining. **(B)** Biacore SPR analysis of Cath B and OC. Cath B was immobilized on a CM5 chip, and different concentrations of OC passed through the chip in 60 s. The curves indicate the potential binding between OC and Cath B. **(C)** the KD value for Cath B and OC binding was calculated from Biacore SPR data. **(D)** and E ITC measurement of Cath B and OC was conducted at 25°C. **(D)** The raw ITC data for injecting OC into Cath B protein. **(E)** The binding curve was integrated by a one-site binding model. **(F)** Inhibitory effect of OC on Cath B protease activity. Cath B was incubated with different concentrations of OC, and the inhibition curve was obtained and calculated using fluorescence intensity.

HSP70s have been proposed to promote cancer cell resistance to adverse microenvironments, such as hypoxia, glucose starvation and chemotherapeutic stresses, which are common features in malignant progression (Shu and Huang, 2008). In addition, HSP70s are highly related to apoptosis, autophagy and metastasis (Budina-Kolomets et al., 2014; Budina-Kolomets et al., 2016). Although our previous studies have indicated that OC partially inhibits autophagic flux by attenuating the Cath B and Cath D proteins (Lao et al., 2014), its detailed mechanism remains to be investigated. As Cath B is an essential protease in the lysosome and plays a vital role in autophagy, we then conducted further studies to evaluate their binding activities using biophysical assays.

### Verifying the Potential Binding of Oblongifolin C to HSPA8 and Cath B

To confirm the potential binding of OC and HSPA8, we performed several protein and small molecular binding assays. First, we performed a Surface Plasmon Resonance (SPR) as a SPR assay is a classical method to confirm protein and small molecule binding. To perform the SPR assay, we subcloned his-tagged HSPA8 gene into pET-15b vector via *Nco*I and *BamH*I cloning site (**Supplementary Figure S6**). As shown in Figure 2A, his-tag HSPA8 exhibited uniform bands by Coomassie blue staining at a molecular weight between 60 and 80 KDa. We then examined the binding affinity of purified HSPA8 and OC in Biacore. As shown in Figure 2B, increasing the concentration of OC caused the binding curve to rise, indicating that the binding of OC to HSPA8 has occurred. The binding affinity was plotted, and the calculated KD value was 11.8 µM (Figure 2C). We also performed an Isothermal titration calorimetry (ITC) assay to measure its binding affinity in a solution. As shown in Figures 2D,E, OC bound to the HSPA8 protein with a binding affinity of 6.2 µM △H was −7.07 kcal/mol, △G is −7.11 kcal/mol, T△S was 0.043 kcal/mol (**Supplementary Table S3**), the KD value was consistent with that of SPR data. Next, we conducted the Thermal Shift assay to examine the effect of OC on HSPA8 stability. The thermal shift assay indicated that the melting curve Tm of HSPA8 unexpectedly decreased after binding with OC as shown in Figure 2F. Compared with the positive control PES that did not shift the HSPA8 Tm value, OC lowered the melting temperature (∼3°C), which disagreed with our ITC results. Various reasons may affect ligands to lower the protein melting temperature, such as some ligands may bind to the unfolded state of protein, or ligands may compete with dyes for binding to the hydrophobic amino acids of protein, resulting in a decrease in the fluorescence signal, which can be observed as a Tm value decrease (Cimmperman et al., 2008; Andreotti et al., 2015).

Cath B, a protease found in the lysosome, plays an important role in lysosomal protein degradation. We then evaluated the binding affinity between OC and Cath B using similar approaches. The Cath B protein used in the Biacore SPR assay was purified, and its purity was evaluated by Coomassie blue staining (Figure 3A). As shown in Figures 3B,C, the response unit showed a dose-dependent response to the OC concentration. Its binding affinity was 39.3 µM, lower than that of OC to HSPA8 (KD, 8.9 µM), suggesting that OC binds weakly to Cath B. We then performed an ITC assay to measure its binding affinity in a solution. We used OC to titrate the Cath B protein. The KD value was 25.6 µM (Figures 3D,E). The thermodynamic parameters of the OC titrate Cath B protein are summarized in **Supplementary Table S3**. Active Cath B cleaves proteins in lysosomes, and inhibition of Cath B suppresses the cleavage activity. Therefore, cathepsin B activity was assayed to examine the inhibitory effects of OC on Cath B (**Supplementary Figure S7** for the description method). OC inhibited Cath B activity with an IC_50_ of 31.9 µM (Figure 3F) consistent with our previous findings, hence we concluded that OC inhibited Cath B activity (Lao et al., 2014).

**FIGURE 4:**
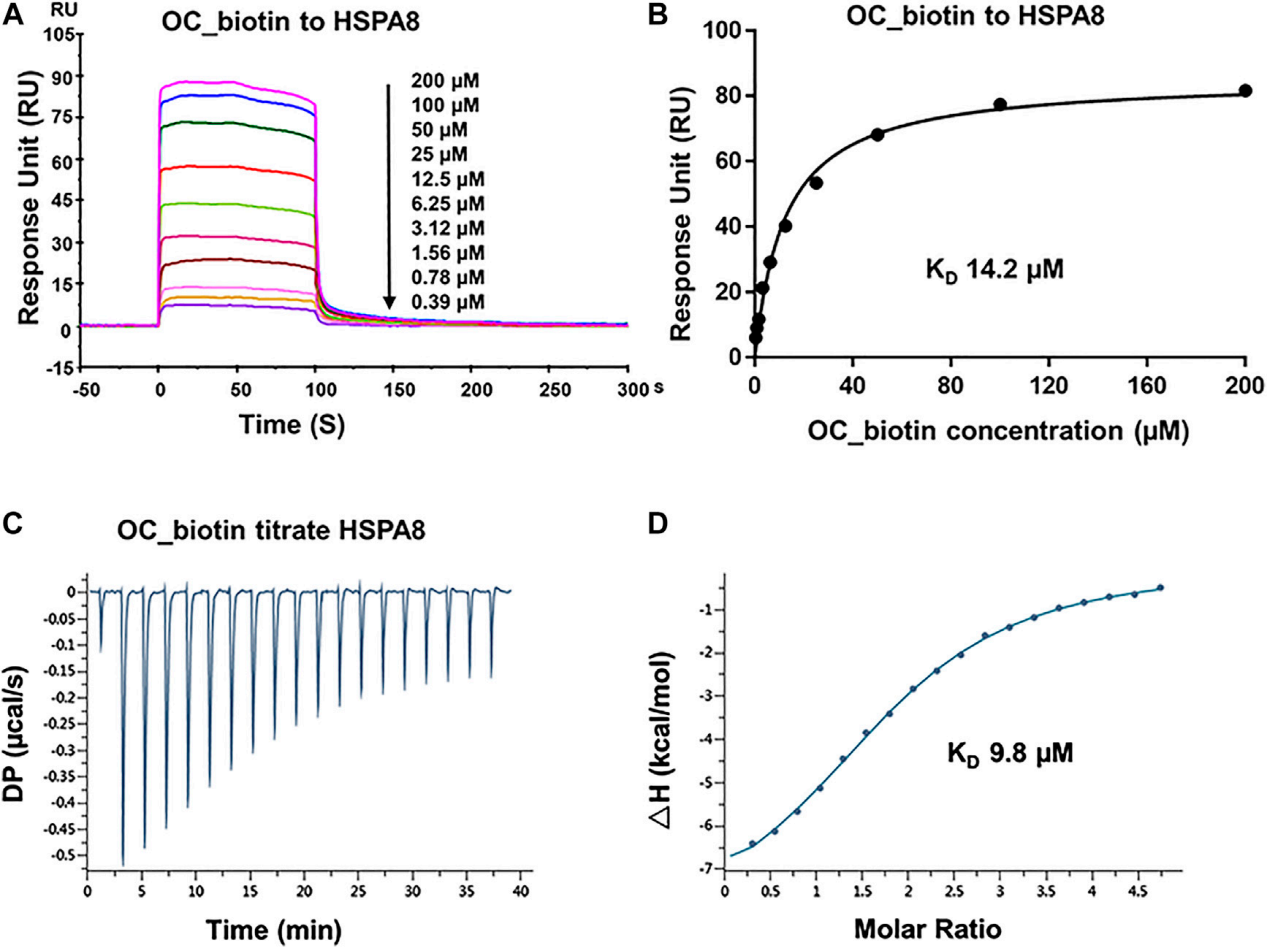
Verifying the potential binding of HSPA8 with OC_biotin. **(A)** Biacore SPR analysis of HSPA8 and OC_biotin potential binding. HSPA8 was immobilized on the CM5 chip and different concentrations of OC_biotin were passed through the chip for 60 s. The curves indicated the potential binding between OC_biotin and HSPA8. **(B)** The KD value was calculated from Biacore SPR data. **(C)** ITC measurement of HSPA8 and OC_biotin was carried out at 25°C. The raw ITC data for injecting OC_biotin into the sample cell containing HSPA8 protein. The reaction heat of OC_biotin and protein binding were expressed as differential power (DP) between the reference and sample cells. **(D)** Experimental data are expressed with solid dots and fitted to a binding curve by a one-site binding model.

**FIGURE 5:**
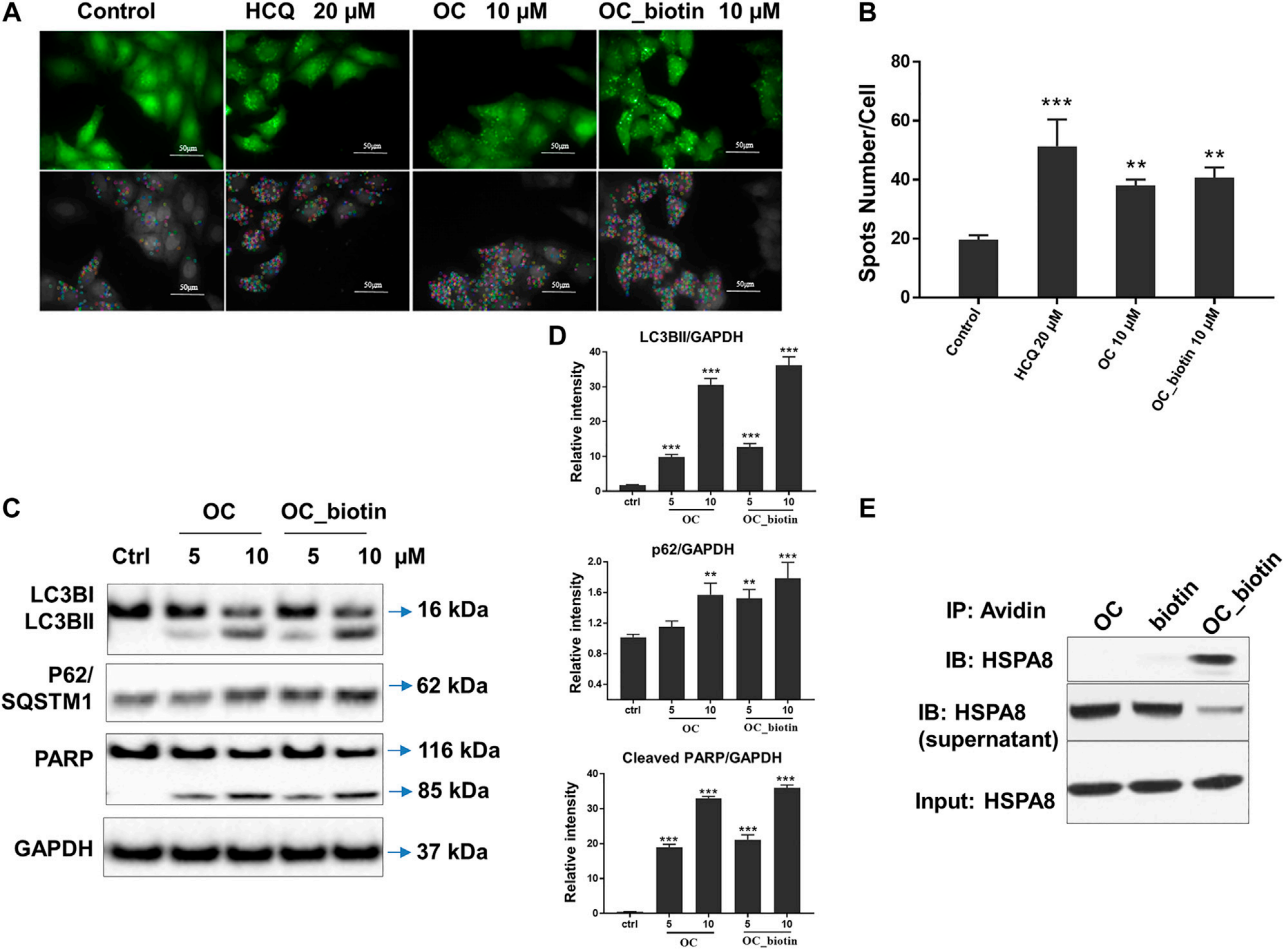
Verifying the potential binding between OC_biotin and HSPA8 in cells. **(A)** OC_biotin or OC induced GFP-LC3 puncta in HeLa cells. HeLa cells with stable expression of GFP-LC3 were treated with 10 µM OC or 10 µM OC_biotin for 6 h, and GFP fluorescence was detected and analyzed using a high content screening system. Scale bar, 50 µm. **(B)** Statistical analysis of the number of GFP puncta formed per cell. Data are expressed as mean ± standard deviation of three independent experiments compared with control (**p < 0.01, ***p < 0.001 vs. control). **(C)** Western blot of LC3B, p62, and PARP. HeLa cells were treated with 10 µM OC or 10 µM OC_biotin for 24 h. The cells were lyzed using RIPA buffer and the protein expression level was analyzed by western blot. **(D)** Statistical analysis on the relative intensity of LC3BII, P62, cleaved PARP vs. GAPDH. GAPDH protein was used as loading control. Data are expressed as mean ± standard deviation of three independent experiments (*p < 0.05, **p < 0.01, ***p < 0.001 vs. control). **(E)** Immunoprecipitation of HSPA8 and OC_biotin. OC_biotin was incubated with avidin beads followed by immunoprecipitation and western blot. Upper lane: pulldown proteins eluted from avidin beads; middle band: proteins from supernatant after immunoprecipitation; lower band: input HSPA8 protein as control.

As shown in SPR and ITC data, the binding affinity of OC to Cath B was weaker than that of OC to HSPA8, justifying our selection of HSPA8 for further studies. To evaluate whether OC potentially binds to HSPA8 in cell lysates, we synthesized OC_biotin (Wang et al., 2016; **Supplementary Figure S8**) and performed a pulldown assay using avidin beads. We first examined whether OC_biotin has the same binding affinity with that of OC to HSPA8 using SPR and ITC assays. As shown in Figures 4A,B, the SPR data showed that the OC_biotin bound to the HSPA8 protein with a binding affinity of 14.2 µM, the ITC data was 9.8 µM (Figures 4C,D); thermodynamic parameters are summarized in **Supplementary Table S3**. Biotin was used as a negative control for SPR and ITC assays (**Supplementary Figure S9**, which indicated that biotin could not bind to HSPA8). Hence, the compound modified by biotin did not affect its binding affinity. We then examined the anticancer activities of OC_biotin and OC in several cancer cell lines. As shown in **Supplementary Table S4**, OC_biotin exerted similar anti-proliferative activity to that of OC in multiple cancer cell lines, with IC_50_ ranging from 5 to 20 µM. In addition, OC_biotin showed similar effects on autophagy and apoptosis in cells. By using HeLa cells that stably expressed GFP-LC3, we measured the GFP-LC3 puncta increment upon OC, OC_biotin and Hydroxychloroquine (HCQ) treatment. As shown in Figures 5A,B, 10 µM OC and OC_biotin resulted in GFP-LC3 puncta formation. Western blot analysis indicated that OC and OC_biotin increased LC3-II/LC3-I ratio, p62/SQSTM1 expression level and induced PARP cleavage, suggesting that OC_biotin has similar bioactivities as OC in cells (Figure 5C). Avidin beads were used to perform the OC_biotin immunoprecipitation assay with cell lysates. Consistent with the SPR and ITC assays, we pulled down HSPA8 using cell lysates. As shown in Figure 5E, HSPA8 was pulled down by OC_biotin, and biotin was used as the negative control. The SPR and ITC assays collectively suggest that OC potentially bind to the HSPA8 protein, while the immunoprecipitation assay implies that HSPA8 potentially binds to OC_biotin in cells.

### Oblongifolin C Inhibits HSPA8 Translocation in Cancer Cells

**FIGURE 6:**
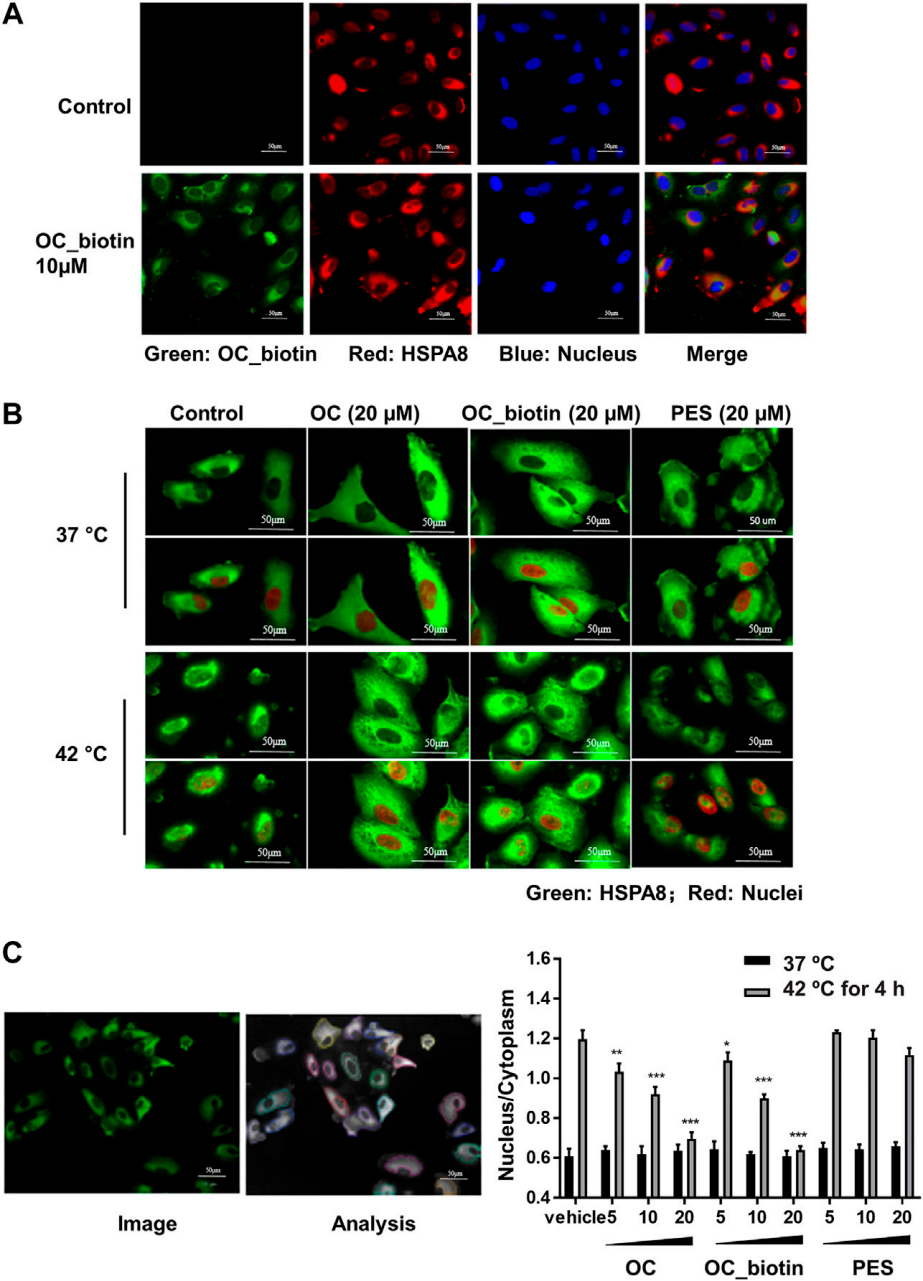
OC and OC_biotin inhibit HSPA8 translocation in cells. **(A)** Colocalization of OC_biotin with HSPA8. A549 cells were treated with 10 µM OC_biotin or DMSO for 1 h. Cells were fixed with 4% paraformaldehyde and stained with streptavidin and HSPA8 antibodies. Cell nuclei were stained by DAPI. Fluorescent images were acquired using a high content screening system. **(B)** OC or OC_biotin blocked HSPA8 translocation following stimulation at 42°C. A549 cells were pretreated with 20 µM OC, 20 µM OC_biotin, or 20 µM PES for 1 h, then incubated at 37°C or 42°C for 4 h. The cells were then fixed and stained with HSPA8 antibody and DAPI. **(C)** Statistical analysis on translocation of HSPA8 from the cytosol to the nucleus. A549 cells were treated with different concentrations of OC, OC_biotin, or PES. The cells were fixed and stained with the HSPA8 antibody. The intensity ratio of the nucleus and cytosol was automatically calculated by a high content screening system. Data are expressed as mean ± standard deviation (*n* = 3, *p < 0.05, **p < 0.01, ***p < 0.001 vs. vehicle control). Scale bar, 50 µm.

The HSP70 family of proteins are chaperones that maintain cell viability in adverse environments, including hypoxia, starvation, and anticancer drug-induced stress. Activation of HSP70 family members is closely associated with tumor transformation and decreased chemotherapy efficacy (Shu and Huang, 2008). We focused on the detailed mechanisms of OC and OC_biotin in regulating HSPA8 in cancer cells. First, we performed immunofluorescent staining to examine the localization of OC_biotin in cells. After being treated with 10 µM OC_biotin for 1 h, OC_biotin was mainly detected in the cytosol of A549 cells. HSPA8 and OC_biotin co-staining indicated that they co-localized in the cytosol under normal culture conditions (Figure 6A). HSP70 translocates from the cytosol to the nucleus under stress condition, contributing to its protective effects. Thus, we investigated whether OC could inhibit HSPA8 translocation in response to stress conditions. As shown in Figures 6B,C, after A549 cells were treated with 4 h heat shock at 42 °C, the nucleic fraction of HSPA8 was significantly increased. In addition, the supplementation of OC or OC_biotin efficiently reduced the HSPA8 concentrations in the nucleic fraction in a dose dependent manner, while PES did not have similar effects. As a HSP70 inhibitor, PES triggers intrinsic apoptosis pathway that consequently retarded proliferation of cervical cancer Hela cells (Liu et al., 2017) as well as induces programmed cell death (PCD) in primary effusion lymphoma (PEL) BC3 and BCBL1 cells. The PCD effect of PES was resultant from promoting lysosome membrane permeability, releasing cathepsin D from lysosome into cytosol, cleaving of Bid, depolarizing mitochondrial and nuclear translocation of apoptosis-activating factor (Granato et al., 2013). As contrary to OC, PES did not impair the nuclear translocation of HSPA8, indicating that the mechanism of OC may differ from PES.

**FIGURE 7:**
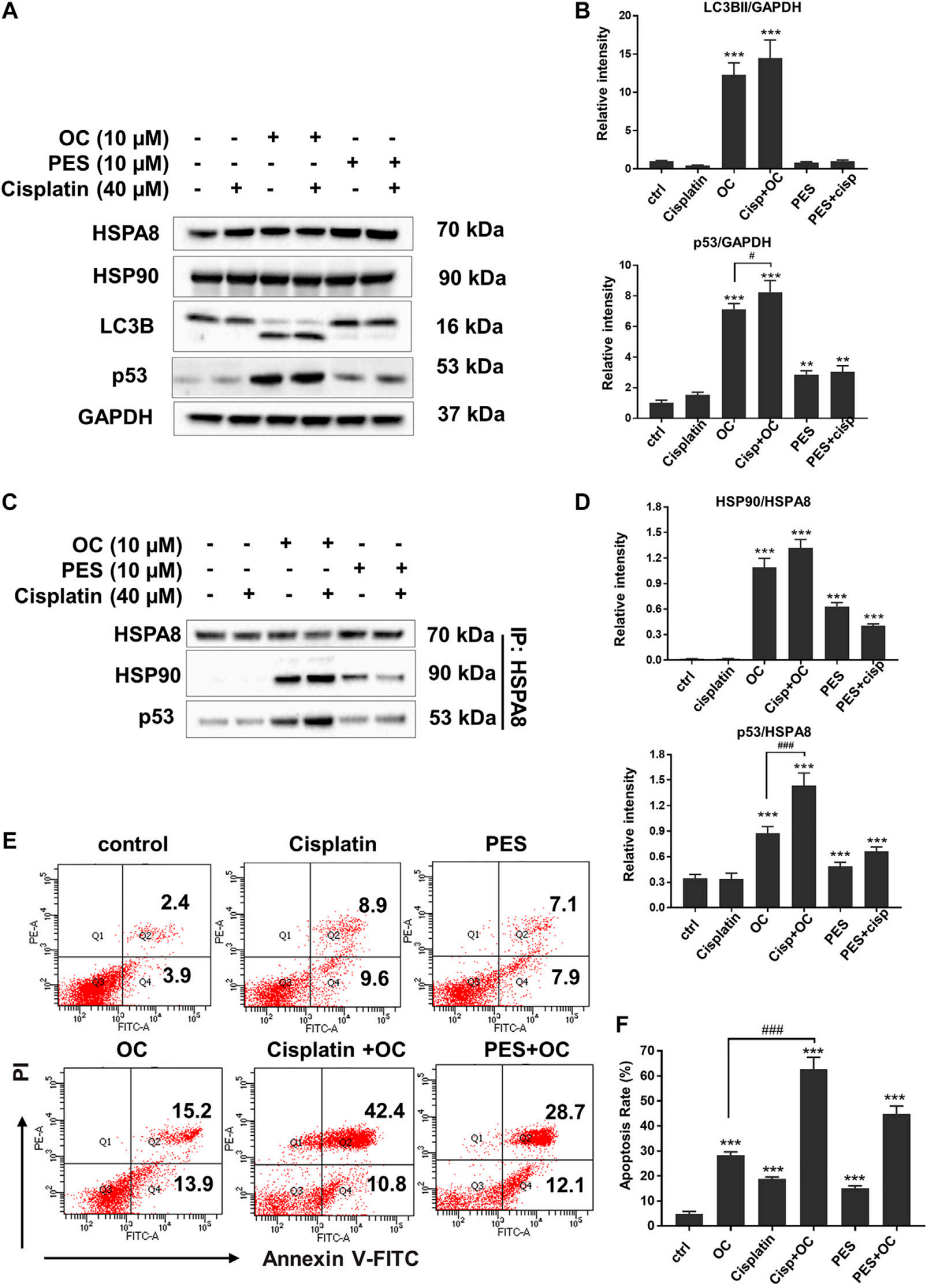
OC promotes apoptosis upon cisplatin treatment. **(A)** OC increased p53 expression level when co-treated with cisplatin. A549 cells were treated with the combination of 10 µM OC, 40 µM cisplatin, and 10 µM PES for 24 h. Western blot analysis showed the changes in p53 and LC3B cleavage. **(B)** Statistical analysis on the relative intensity of LC3BII and P53 vs. GAPDH. GAPDH protein was used as loading control. Data are expressed as mean ± standard deviation of three independent experiments (*p < 0.05, **p < 0.01, ***p < 0.001 vs. control; ^#^ *p* < 0.05 vs. OC group). **(C)** OC enhanced the binding between HSPA8, HSP90, and p53. A549 cells were treated with the combination of 10 µM OC, 40 µM cisplatin, or 10 µM PES for 24 h. Cell lysates were immunoprecipitated with HSPA8, and western blot analysis was performed for HSPA8, HSP90, and p53. **(D)** Statistical analysis on the relative intensity of HSP90 and P53 vs. HSPA8. HSPA8 protein was used as loading control. Data are expressed as mean ± standard deviation of three independent experiments (*p < 0.05, **p < 0.01, ***p < 0.001 vs. control; ^###^ *p* < 0.001 vs. OC group). **(E,F)** OC potentiated cell death upon cisplatin treatment. A549 cells were treated with the combination of 10 µM OC, 40 µM cisplatin, or 10 µM PES for 24 h. The cells were collected and stained with Annexin V and PI for subsequent flow cytometry analysis. F shows the results of statistical analysis of apoptotic cells. Data are expressed as mean ± standard deviation of three independent experiments (*p < 0.05, **p < 0.01, ***p < 0.001 vs. control; ^###^ *p* < 0.001 vs. OC group).

HSP family member proteins, including HSP70 and HSP90, are essential in protecting malignant cells from death under multiple microenvironmental stresses, such as hypoxia, nutrient deprivation, and chemotherapy stress. Our previous study has demonstrated that OC sensitizes cancer cells to apoptosis during nutrient starvation by partially inhibiting autophagic flux. Here, we investigated whether OC could influence cell response to therapeutic agents as well as how HSP proteins and their binding protein p53 change during OC and cisplatin treatment. We first examined the changes in certain key proteins upon cisplatin, OC, and PES treatment. As shown in Figure 7A, HSPA8 and HSP90 did not significantly change after 24 h of treatment. The autophagy marker LC3-II/LC3-I ratio increased in the OC and OC-cisplatin treatment groups, suggesting that OC efficiently regulates autophagic signals. Interestingly, we found that OC significantly increased p53 expression level, the supplementation of OC and cisplatin efficiently amplified p53 expression level, while p53 expression level hardly had any change during cisplatin treatment. PES treatment also mildly increased p53 expression level (Figure 7A). Because the interaction between HSP70, HSP90, and p53 dominantly sustain the activity of p53 protein (Muller et al., 2004; Walerych et al., 2009), we further investigated how OC affects their binding with or without cisplatin treatment. HSPA8 immunoprecipitation showed that OC could strengthen the interaction between HSPA8, HSP90 and p53. As shown in Figure 7C, cisplatin treatment did not affect the binding between these proteins. However, OC promoted binding of HSP90 and HSPA8 as well as p53 and HSPA8, and the supplementation of OC and cisplatin strengthened their interactions. In addition, PES slightly affected the interactions between the above mentioned proteins. We then examined whether OC could promote apoptosis upon cisplatin treatment using PI/Annexin V double staining in flow cytometry. A549 cells were treated with 10 µM OC or/and 40 µM cisplatin for 24 h before the flow cytometry assay and Figures 7E,F show that OC significantly promotes apoptosis in cisplatin-treated cells.

**FIGURE 8:**
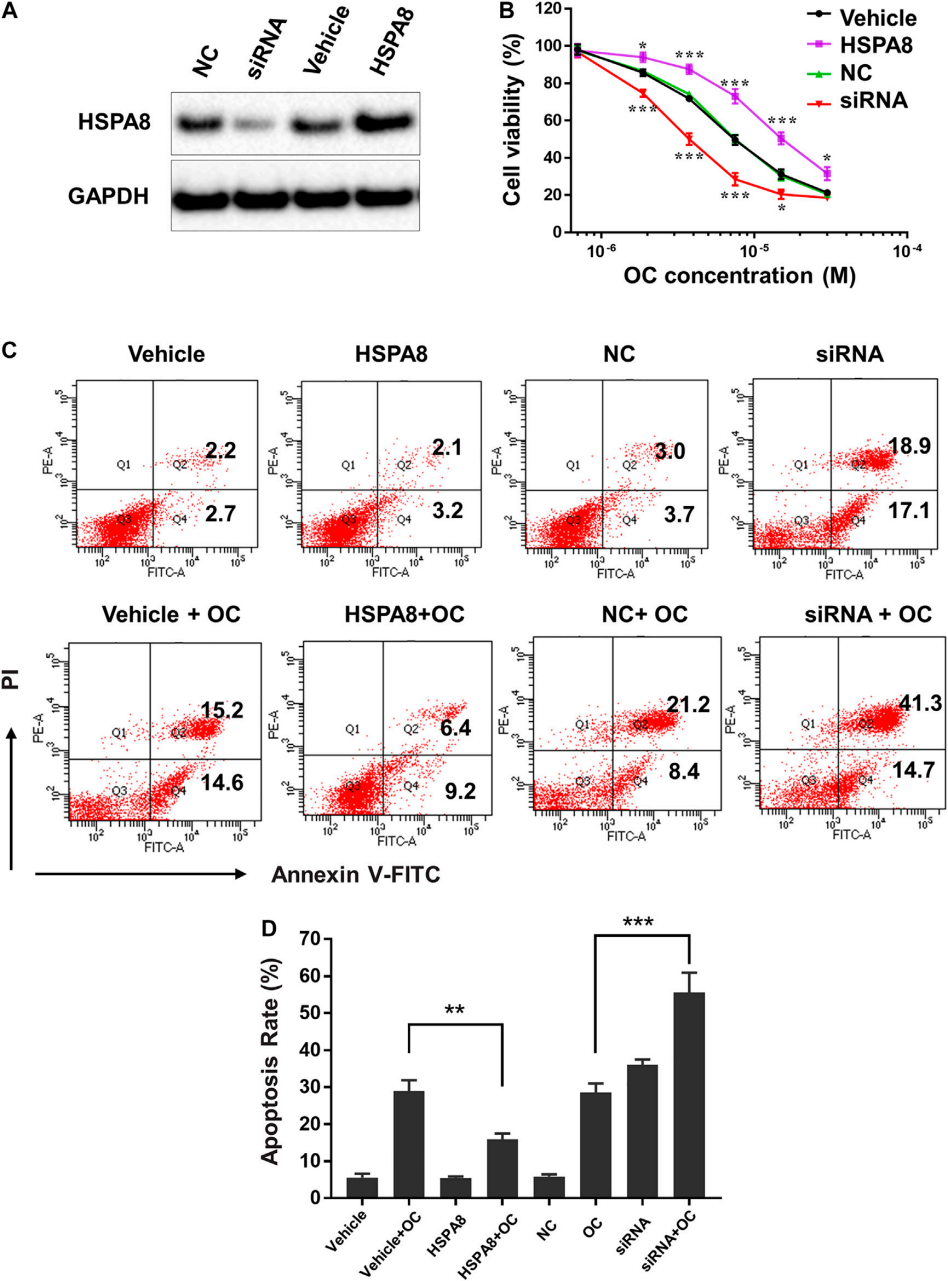
OC induces apoptosis of A549 cells by regulating HSPA8 protein. **(A)** Detection of the HSPA8 protein expression level in A549 cells by western blot analysis after 48 h of transfection, GAPDH was used as loading control. A549 cells were transfected with small interfering RNA (siRNA) and pCDNA_HSPA8 plasmids respectively for 48 h siRNA control (NC) and pCDNA3.1 vector (Vehicle) as a control. **(B)** The effects of OC on A549 cell viability after treatment with HSPA8 siRNA or pCDNA_HSPA8 plasmid. The cells were transfected with siRNA or plasmid for 48 h and OC was supplemented at the indicated concentrations for 72 h. Cell viability was then detected using the CCK-8 Assay. Data are expressed as mean ± standard deviation of three independent experiments (*p < 0.05, **p < 0.01, ***p < 0.001 vs. control). **(C)** OC induces apoptosis of A549 cells by targeting HSPA8 protein. A549 cells were transfected with HSPA8 siRNA or plasmid for 48 h, 10 µM OC was supplemented for another 24 h. The cells were collected and stained with Annexin V and PI for flow cytometry analysis. **(D)** The result of statistical analysis of apoptotic cells. Data are expressed as mean ± standard deviation of three independent experiments (**p < 0.01, ***p < 0.001 vs. OC control group).

Considering that HSP70 is highly expressed in various cancer cells, we examined whether OC still affects cell apoptosis following HSPA8 knockdown by small interfering RNA (siRNA) or overexpression by plasmid. As shown in Figure 8A, the HSPA8 expression level was significantly reduced by siRNA after 48 h transfection. Transfecting HSPA8 plasmid increased the expression level of HSPA8 in A549 cells (Figure 8A). We then used the CCK-8 assay to assess the cytotoxicity of OC on A549 cells transfected with HSPA8 siRNA and HSPA8 plasmid. As shown in Figure 8B, OC decreased the cell viability rate in a dose dependent manner. The IC_50_ value of OC was 7.4 ± 1.18 µM in vehicle and NC control groups. Effect of OC inducing A549 cell death was enhanced by transfecting HSPA8 siRNA and attenuated by HSPA8 overexpression, with respective IC_50_ value of 3.6 ± 1.26 µM and 15.3 ± 2.34 µM. Consistent with this result, a flow cytometry assay suggested that OC sped the apoptosis rate in HSPA8 knockdown A549 cells up, while slowing the apoptosis rate in HSPA8 overexpressed cells down (Figures 8C,D), collectively indicating that HSPA8 is one of the potential target proteins of OC.

In summary, the present study provides evidence that the natural compound OC potentially binds to HSPA8 and Cath B proteins, subsequently suppressing their functions in cancer cells. These data indicated that OC could act on multiple proteins that are related to several important signaling pathways, including apoptosis, autophagy and drug resistance.

## Discussion

OC is a natural compound with favorable biological effects such as markedly slowing down the growth of xenograft tumors of human melanoma MDA-MB-435 cells; it has reduced toxicity *in vivo* compared to chemotherapy etoposide (Feng et al., 2012); and has high and preferential cytotoxicity on colon cancer cells (Xu et al., 2010; Li et al., 2017). However, elucidating the mechanism of action (MOA) of natural compounds has bottlenecked the development of novel drugs from traditional medicines. Recognizing the pressing need of identifying protein targets of natural compounds to guide efficacy improvement, this present study identified the protein targets by applying an unlabeled technique to pull down the potential binding proteins of OC from cell lysates. As expected, OC potentially bound to multiple proteins in cancer cells, including HSPA8 and Cath B, which partially explains the complicated MOA of OC in several important signaling pathways, such as autophagy and apoptosis. We confirmed the potential binding between OC and HSPA8 or Cath B using biophysical approaches, including Biacore SPR and ITC assays. OC blocked the nuclear translocation of the HSPA8 protein (Figures 6B,C), contrarily PES the HSPA8 inhibitor did not have this inhibitory effect. These findings asserted that OC might have acted differently on nuclear translocation of HSPA8 than PES, which requires further investigation. Apart from differed effects on nuclear translocation of HSPA8, OC, and PES similarly increased the expression level of p53, regulated interactions of HSPA8, HSP90, and p53 (Figures 7A,C) and promoted apoptosis in cisplatin-treated cells (Figure 7E). Furthermore, we found that OC inhibited HSPA8 nuclear translocation in cancer cells, which then suppressed the protective effects of HSPA8 under certain stresses. Therefore, OC plausibly inhibits autophagic flux by suppressing the translocation of HSPA8, necessitating future studies for substantiation. OC significantly increased the p53 expression level and accelerated the apoptosis rate in cisplatin-supplemented cells. Altogether, our findings highlight that OC sensitizes A549 cells to starvation-induced apoptosis and signify the potential of OC in cancer therapy when combined with other chemotherapeutics. More studies are required to confirm whether OC synergize chemotherapy cisplatin in reducing levels of intranuclear HSPA8, which may subsequently promote an apoptosis effect.

In summary, we emphasize the findings on the inhibitory effect of OC on HSPA8 in cancer cells under heat shock stress, by inhibiting the translocation of HSPA8. The mechanism of action of OC is currently still under study with other possible mechanisms of action and targets awaiting discovery. We previously identified OC as a potent apoptosis inducer in cervical HeLa cells through a caspase-dependent pathway that involved Bax translocation, cytochrome c release, caspase-3 activation, chromosome fragmentation, followed by caspase-8 activation, Bid cleavage, and eventually cell death (Feng et al., 2012). OC also inhibited pancreatic cancer cell proliferation by inducing G0/G1 arrest as well as induced apoptosis *via* downregulating Src (Li et al., 2018).

Drug development from natural compounds requires chemical synthesis and structure modification to increase drug efficacy such as pharmacokinetics and absorption. The anti-malaria drug artemether (modified from artemisinin) is an example of this process (Tu, 2011). Although we attempted to modify OC to increase its inhibitory effect on c-Met kinase, most of the variants exhibited lower activities (Wang et al., 2016). With the development of structural biology, researchers can identify protein structures and design small molecules based on a protein and a small molecular complex structure. We sought to crystalize the full length HSPA8 protein bound to OC but were unsuccessful (data not shown). Our future studies will focus on how OC changes the protein structure by crystalizing the ATP binding domain and Lid-Tail domains of HSPA8 with OC or OC analogs, to compare the protein conformation changes.

The present study also raised several interesting mechanistic questions. First, does inhibition of HSPA8 translocation by OC correlate with inhibition of autophagy? Leu et al. reported that HSP70 inhibition impairs protein clearance, followed by cell death (Leu et al., 2011). Later, Jäättelä’s group extensively studied the functional role of lysosomal HSP70 on the maintenance of the lysosome structure and cell death (Petersen et al., 2013; Hamalisto and Jaattela, 2016). Therefore, it is of interest to investigate whether HSPA8 plays a role in the lysosome structure and autophagy. Second, does inhibition of HSP70 or Cath B attenuate cancer cell metastasis? Recent studies indicate that HSP70 is involved in tumorigenesis and metastasis (Gong et al., 2015). In addition, cathepsin family members were shown to play multiple roles in cancer inhibition (Aggarwal and Sloane, 2014). Further development of OC and its variants as more specific protein inhibitors, will therefore promote the preclinical study of related signaling pathways.

## Data Availability Statement

The datasets presented in this study can be found in online repositories. The names of the repository/repositories and accession number(s) can be found in the article/**Supplementary Material**.

## Author Contributions

The research was designed by CG and HX. LH, DX, JZ, WNN, and WF conducted the experiments. MW, GX, and HS assisted with data analysis. The manuscript was written by LH, YL, ZX, and HX. The study was directed by HZ, CG, and HX.

## Funding

This work was financially supported by the National Natural Science Foundation of China (nos. 81773951, 81803571 and 81503102), the NSFC-Joint Foundation of Yunan Province (U1902213), the Shanghai Education Foundation of SHUTCM (ZYX-CXYJ-011), the Three-year development plan project for Traditional Chinese Medicine: ZY (2018-2020)-CCCX-2001-02, and the Key-Area Research and Development Program of Guangdong Province (2020B1111110003).

## Conflict of Interest

The authors declare that the research was conducted in the absence of any commercial or financial relationships that could be construed as a potential conflict of interest.
