# Supplementary material for: The Natural Compound Oblongifolin C Exhibits Anticancer Activity by Inhibiting HSPA8 and Cathepsin B In Vitro
Source: Front Pharmacol. 2020 Dec 17;11:564833. doi: 10.3389/fphar.2020.564833 (PMC7773843; doi:10.3389/fphar.2020.564833)
Supplement: Supplementary file 1 [file datasheet1.docx]

***Supplementary Material***

**Supplementary Figure S1**

**

**

**Supplementary Figure S1** The structure of OC

**Supplementary Figure S2**
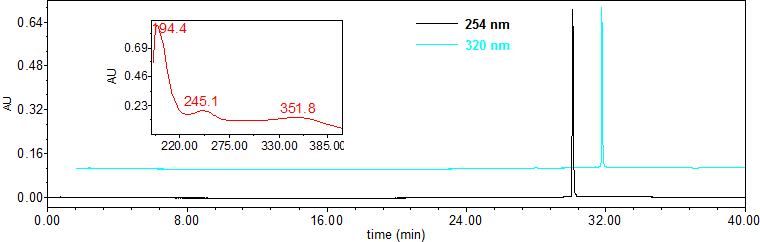


**Supplementary Figure S2** UPLC chromatogram of OC

Column: Waters ACQUITY UPLC® BEH C18 (1.7 μm, 2.1 * 100 mm); mobile phase: (C) acetonitrile and (D) 0.1% formic acid in water; gradient mode: 0–5 min, 32–42% (C); 5–10 min, 42–46% (C); 10–23 min, 46–70% (C); 23–29 min, 70–95% (C); 29–32 min, 95–100% (C); 32–33 min, 100–95% (C); 33–35 min, 95–32% (C); and 35–40 min, 32% (C); detection wavelength: 250 and 320 nm; flow rate: 0.4 mL·min^-1^; the column and autosampler were maintained at 40 °C and 10 °C respectively.

OC was isolated from the fruits of *Garcinia yunnanensis* Hu using the same preparation method as described previously (Wang et al., 2016). Air-dried and powdered plants of *Garcinia yunnanensis* Hu were extracted by refluxing with 95% EtOH (v/v, 3 × 500 L). The combined extracts were evaporated till no alcohol taste remained, and were subsequently diluted in H_2_O and extracted in turn with petroleum ether and EtOAc to obtain the dried petroleum ether- (2.5 kg), EtOAc (3.3 kg), and H_2_O-soluble extracts. The petroleum ether soluble extract was subjected to the passage over a chromatography column (CC) on the MCI (6 L) and were successively eluted with H_2_O (9 L), 95% EtOH (18 L), and EtOAc (12 L). The 95% EtOH-eluting fraction was chromatographed by silica gel CC using a gradient of petroleum ether–acetone (100:0 to 50:50, v/v) and yielded 16 fractions, A–P, by the analysis of their TLC profiles. OC (200 mg) was isolated from fraction E (43 g) by reversed phase C_18_ silica gel CC and preparative HPLC (MeCN–H_2_O, 82:18, with 0.1% formic acid in H_2_O, 20 mL/min). The purity of OC was more than 95% based on UPLC analysis.

**Supplementary Figure S3**
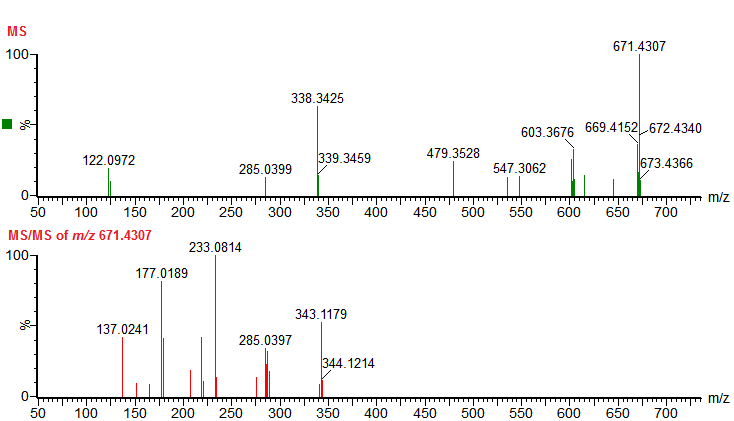


**Supplementary Figure S3** The MS and MS/MS spectra of OC

Mass spectra were obtained using a SYNAPT G2-Si HDMS (Waters Corp., Manchester, UK) equipped with an electrospray ionization source. ESI mass spectra were acquired over the *m/z* 50–1200 range. The desolvation gas was set to 800 L/h at temperature of 400 °C, the cone gas was set to 50 L/h, and the source temperature was set to 120 °C. The capillary voltage was set to 2500 V. Spectra were acquired in continuum and positive mode. Argon was employed as the collision gas. The SYNAPT G2-Si HDMS system was calibrated using sodium formate clusters and operated in resolution mode. Leucine enkephalin was used as a lock mass. OC was analyzed by UPLC-QTOFMS using the data-dependent acquisition mode. The top five ions were selected for MS/MS from a single MS survey scan. The scan time for MS/MS was 0.2 s. The collision energy in the trap cell was ramped from 20 to 45 V. Masslynx 4.1 was used for visualization, processing, and interpretation of MS data.

The compound in our experiment was obtained as a yellow gum, possesses the same molecular formula (C43H58O6) as OC, as determined from HRESIMS [M+H]^+^ ions at m/z 671.4307. The MS/MS spectrum of the compound showed a diagnostic fragment ion at m/z 177.0189 in the positive mode suggesting that it might be a type B PPAP (Zhang et al., 2016).

**Supplementary Figure S4**

**Supplementary Figure S4** The ^1^H NMR spectrum of OC

The ^1^H NMR spectrum indicated the presence of a 1,3,4-trisubstituted benzene ring [*δ*_H_ 7.21 (1H, d, *J* = 2.1), 6.96 (1H, dd, *J* = 8.3, 2.1), and 6.69 (1H, d, *J* = 8.3)], four olefinic protons [*δ*_H_ 5.06 (1H, m), 5.05 (1H, m), 5.01 (1H, m) and 4.87 (1H, m)] and ten methyl groups [*δ*_H_ 1.71 (3H, s), 1.70 (3H, s), 1.67 (3H, s), 1.66 (3H, s), 1.65 (3H, s), 1.63 (3H, s), 1.60 (3H, s), 1.57 (3H, s), 1.65 (3H, s) and 0.82 (3H, s)], which were in accordance with that of OC (Hamed et al., 2006) (Table 1).

**Supplementary Table S1.** The ^1^H NMR data of OC

|  | OC (reference) *^a^* | OC *^b^* |
| --- | --- | --- |
| no | *δ*_H_, mult. (*J* in Hz) | *δ*_H_, mult. (*J* in Hz) |
| 1 |  |  |
| 2 |  |  |
| 3 |  |  |
| 4 |  |  |
| 5 |  |  |
| 6 | 2.07, m; 1.46, t (12.8) | 2.07, m; 1.46, t (12.8) |
| 7 | 1.78, m | 1.78, m |
| 8 |  |  |
| 9 |  |  |
| 10 |  |  |
| 11 |  |  |
| 12 | 7.20, d (2.1) | 2.21, d (2.1) |
| 13 |  |  |
| 14 |  |  |
| 15 | 6.70, d (8.3) | 6.69, dd (8.3) |
| 16 | 6.98, dd (8.3, 2.1) | 6.96, dd (8.3, 2.1) |
| 17 | 2.74, dd (13.0, 9.0); 2.66, m | 2.73, dd (13.8, 8.0); 2.65 (13.8, 4.3) |
| 18 | 4.87, m | 4.87, m |
| 19 |  |  |
| 20 | 1.62, s | 1.63, s |
| 21 | 1.69, s | 1.70, s |
| 22 | 0.82, s | 0.82, s |
| 23 | 1.68, s | 1.67, s |
| 24 | 2.10, m; 1.77, m | 2.10, m; 1.77, m |
| 25 | 5.00, m | 5.01, m |
| 26 |  |  |
| 27 | 1.98, m | 1.98, m |
| 28 | 1.56, s | 1.56, s |
| 29 | 2.54, dd (8.0, 14.0); 2.46, m | 2.53, dd (14.0, 8.8); 2.45, dd (13.9, 5.1) |
| 30 | 5.12, m | 5.12, m |
| 31 |  |  |
| 32 | 1.71, s | 1.71, s |
| 33 | 1.66, s | 1.65, s |
| 34 | 2.06, m | 2.06, m |
| 35 | 5.05, m | 5.05, m*^c^* |
| 36 |  |  |
| 37 | 1.64, s | 1.63, s |
| 38 | 1.57, s | 1.57, s |
| 39 | 1.98, m | 1.98, m |
| 40 | 5.05, m | 5.06, m*^c^* |
| 41 |  |  |
| 42 | 1.67, s | 1.66, s |
| 43 | 1.60, s | 1.60, s |

*^a^* Literature data of OC recorded in methanol-*d*_4_ + 0.1%TFA at 600 MHz (^1^H).

*^b^* Recorded in methanol-*d*_4_ + 0.1%TFA at 600 MHz (^1^H) in our experiment.

*^c^* Data may be interchangeable

**Supplementary Figure S5**


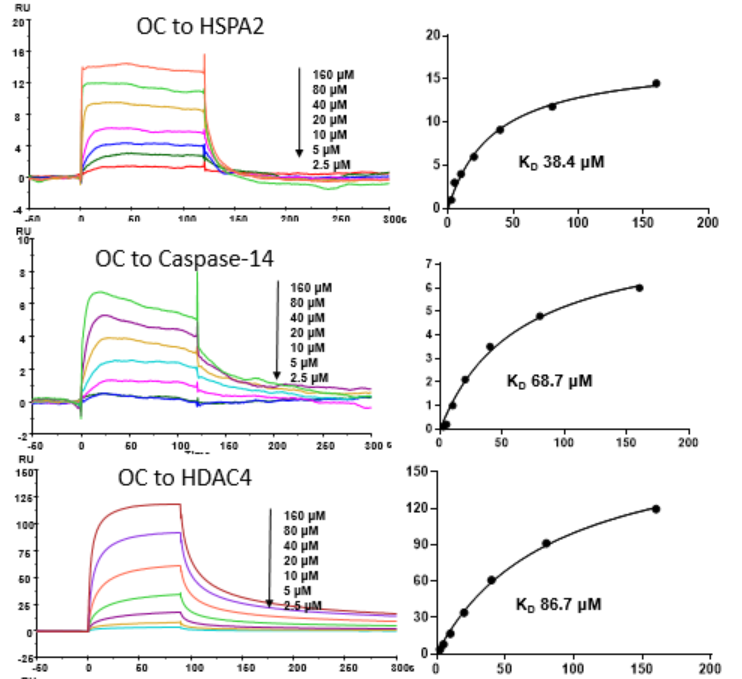


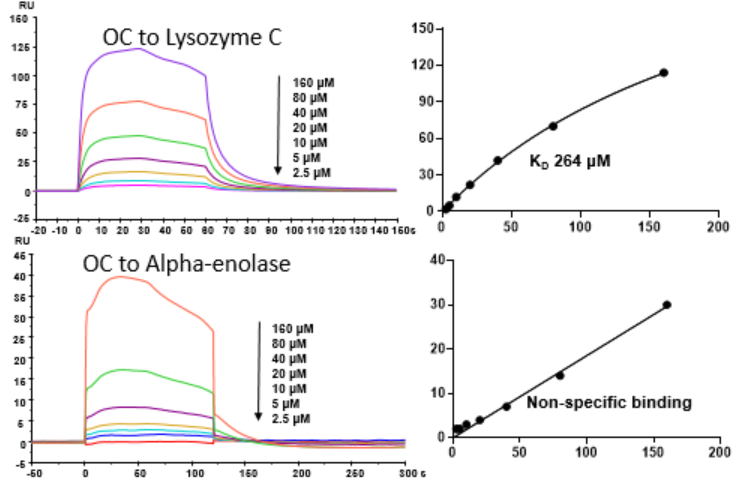


**Supplementary Figure S5** Comparing OC affinity for different proteins

**Supplementary Figure S6**


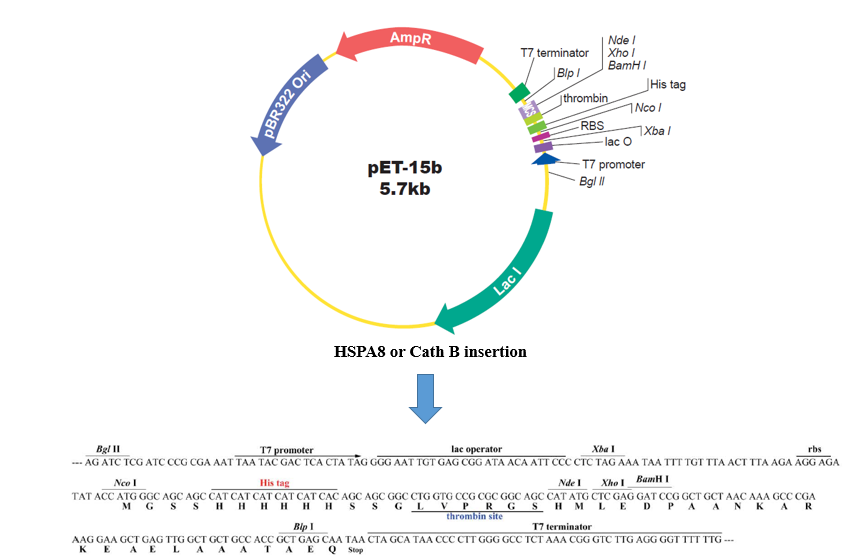


**Supplementary Figure S6** Genetic map of plasmid HSPA8 and Cathepsin B

**Supplementary Figure S7**


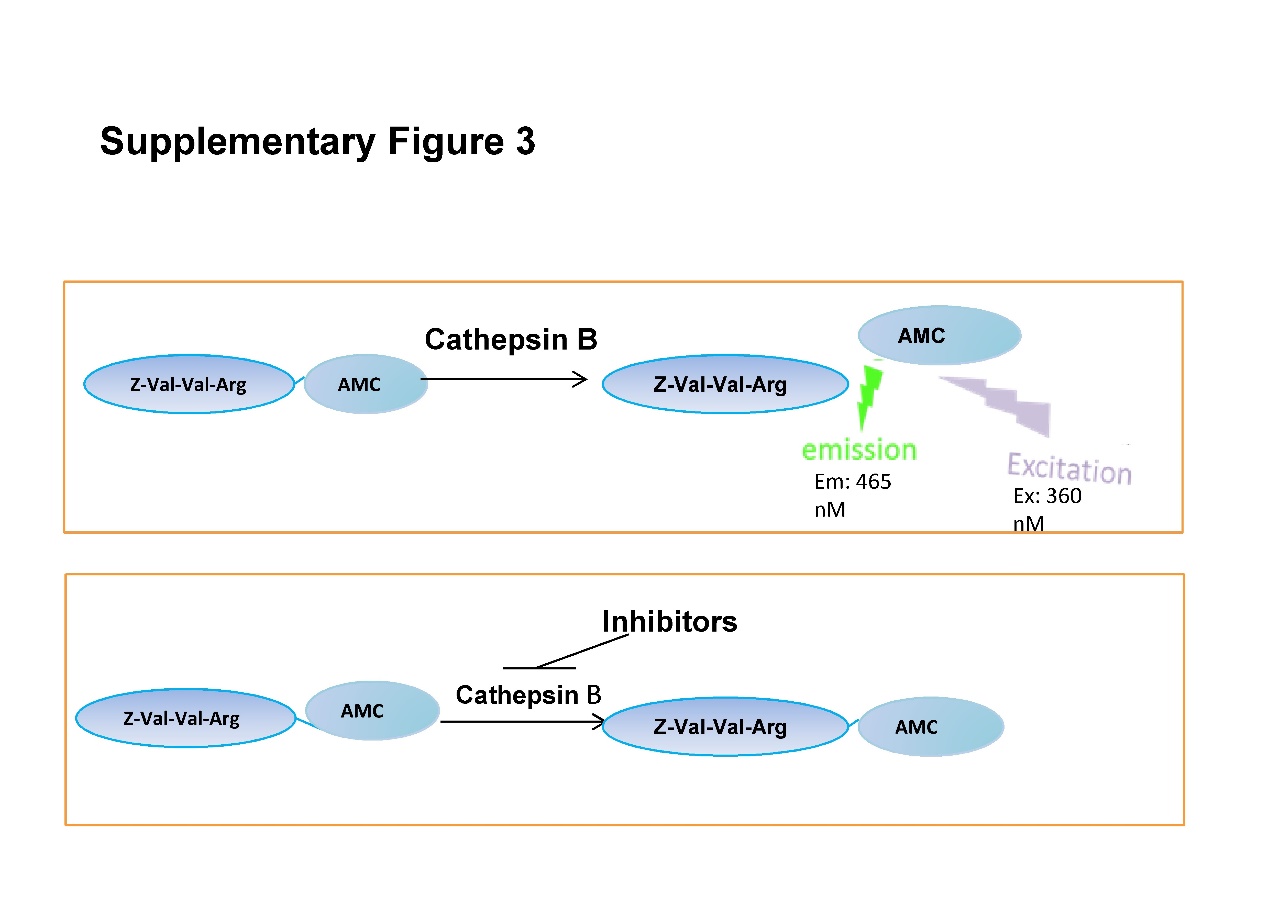


**Supplementary Figure S7** The Cathepsin B activity assay

After incubating different concentrations of OC with Cathepsin B, the enzyme activity and the IC_50_ value were determined.

**Supplementary Figure S8**

**
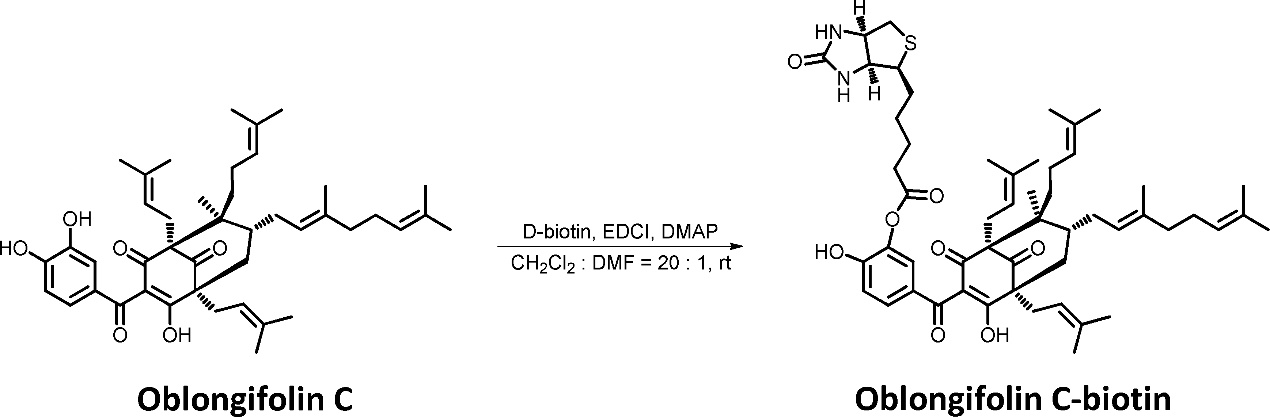
**

**Supplementary Figure S8** Synthesis of OC_biotin

The scheme for the synthesis of OC_biotin is shown in **Figure 7** (Wang et al., 2016). EDCI (9 mg, 0.045 mmol) and DMAP (0.4 mg, 0.003 mmol) were added to a solution containing OC (10 mg, 0.015 mmol) in dry CH2Cl:DMF (5 mL, 20:1), followed by D-biotin (4.2 mg, 0.017 mmol). The reaction mixture was left to stir overnight at room temperature. On the next day, the mixture was diluted with water (5 mL) and extracted with EtOAc (3×5 mL). The resulting organic solution was washed with saturated brine, dried over Na­_2_SO_4_, and concentrated in vacuo. The residue was chromatographed on silica gel (CH2Cl2: MeOH, 10:1) to yield OC_biotin as a pale yellow solid (6 mg, 46%). HRMS (ESI): Calcd. for [M-H]- (C53H71N2O8S) requires m/z 895.5009, found 895.4896. The purity of OC_biotin was >96% based on UPLC analysis (Wang et al., 2016).

**Supplementary Figure S9**
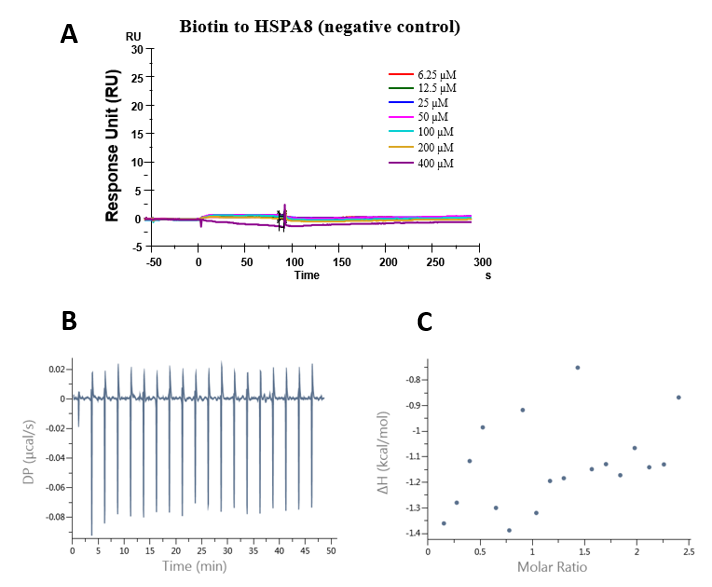


**Supplementary Figure S9** Inability of biotin bind to HSPA8

**A** HSPA8 was immobilized on the CM5 chip and different concentrations of Biotin were passed through the chip for 90 sec. There was no response unit. **B and C** Isothermal titration calorimetry (ITC) measurement of biotin and HSPA8 was conducted at 25 °C. **B** The raw data of ITC for injecting biotin into HSPA8 protein. **C** The binding curve was integrated by a one-site binding model.

**Supplementary Table S3.** OC or OC_biotin titrate protein target

| Small molucular | Protein target | KD (µM) | △H (kcal/mol) | △G  (kcal/mol) | T△S  (kcal/mol) |
| --- | --- | --- | --- | --- | --- |
| OC | HSPA8 | 6.2 | -7.07 | -7.11 | 0.043 |
| OC | Cath B | 25.6 | -9.06 | -6.27 | -2.79 |
| OC_biotin | HSPA8 | 9.8 | -8.59 | -6.83 | -1.75 |

**Supplementary Table S4.** Cytotoxicity of OC and OC_biotin on 8 cell lines

| Cell lines | IC_50_ (µM) | | | | | | | |
| --- | --- | --- | --- | --- | --- | --- | --- | --- |
|  | Hela | A549 | PANC-1 | HL-7702 | PC-3 | MDA-MB-231 | HCT116 | HCT116-DKO |
| OC | 11.03±1.2 | 7.49±0.9 | 17.5±0.9 | 5.16±0.4 | 10.7±0.6 | 15.9±1.1 | 6.15±0.5 | 12.15±0.9 |
| OC_biotin | 6.15±0.7 | 5.62±0.7 | 16.12±1.4 | 3.3±0.3 | 7.5±0.4 | 7.7±0.8 | 3.46±0.3 | 6.82±0.4 |
| Etoposide | 2.93±0.4 | 1.31±0.3 | 22.74±1.7 | 1.42±0.2 | 4.8±0.2 | 4.5±0.4 | 0.5±0.1 | 1.7±0.2 |

**References**

Hamed, W., Brajeul, S., Mahuteau-Betzer, F., Thoison, O., Mons, S.P., Delpec, B., Hung, N.V., Se´Venet, T., and Marazano, C. (2006). Oblongifolins A-D, Polyprenylated Benzoylphloroglucinol Derivatives from Garcinia oblongifolia. *J. Nat. Prod.* 69**,** 774-777.

Wang, L., Wu, R., Fu, W., Lao, Y., Zheng, C., Tan, H., and Xu, H. (2016). Synthesis and biological evaluation of Oblongifolin C derivatives as c-Met inhibitors. *Bioorg Med Chem* 24**,** 4120-4128.

Zhang, H., Zheng, D., Li, H.-H., Wang, H., Tan, H.-S., and Xu, H.-X. (2016). Diagnostic filtering to screen polycyclic polyprenylated acylphloroglucinols from Garcinia oblongifolia by ultrahigh performance liquid chromatography coupled with ion mobility quadrupole time-of-flight mass spectrometry. *Analytica Chimica Acta* 912**,** 85-96.
